# An *In Vitro* Mechanism Study on the Proliferation and Pluripotency of Human Embryonic Stems Cells in Response to Magnesium Degradation

**DOI:** 10.1371/journal.pone.0076547

**Published:** 2013-10-17

**Authors:** Thanh Yen Nguyen, Chee Gee Liew, Huinan Liu

**Affiliations:** 1 Department of Bioengineering, University of California Riverside, Riverside, California, United States of America; 2 Stem Cell Center, University of California Riverside, Riverside, California, United States of America; 3 Materials Science and Engineering Program, University of California Riverside, Riverside, California, United States of America; University of California, San Diego, United States of America

## Abstract

Magnesium (Mg) is a promising biodegradable metallic material for applications in cellular/tissue engineering and biomedical implants/devices. To advance clinical translation of Mg-based biomaterials, we investigated the effects and mechanisms of Mg degradation on the proliferation and pluripotency of human embryonic stem cells (hESCs). We used hESCs as the *in vitro* model system to study cellular responses to Mg degradation because they are sensitive to toxicants and capable of differentiating into any cell types of interest for regenerative medicine. In a previous study when hESCs were cultured *in vitro* with either polished metallic Mg (99.9% purity) or pre-degraded Mg, cell death was observed within the first 30 hours of culture. Excess Mg ions and hydroxide ions induced by Mg degradation may have been the causes for the observed cell death; hence, their respective effects on hESCs were investigated for the first time to reveal the potential mechanisms. For this purpose, the mTeSR®1 hESC culture media was either modified to an alkaline pH of 8.1 or supplemented with 0.4–40 mM of Mg ions. We showed that the initial increase of media pH to 8.1 had no adverse effect on hESC proliferation. At all tested Mg ion dosages, the hESCs grew to confluency and retained pluripotency as indicated by the expression of OCT4, SSEA3, and SOX2. When the supplemental Mg ion dosages increased to greater than 10 mM, however, hESC colony morphology changed and cell counts decreased. These results suggest that Mg-based implants or scaffolds are promising in combination with hESCs for regenerative medicine applications, providing their degradation rate is moderate. Additionally, the hESC culture system could serve as a standard model for cytocompatibility studies of Mg *in vitro,* and an identified 10 mM critical dosage of Mg ions could serve as a design guideline for safe degradation of Mg-based implants/scaffolds.

## Introduction

Various biomaterials have been explored with different stem cell types for enhanced tissue regeneration [1], [2], [3], [4]; however, integration of magnesium (Mg) scaffolds with human pluripotent stem cells remains unexplored despite its great potential. Mg combines the inherent mechanical strength and conductivity of metals with biodegradability and biocompatibility in the human body, making it promising for the use in biomedical implants and scaffolds. For instance, Mg is currently being explored for bone implants because it has a high strength-to-mass ratio and an elastic modulus of 45 GPa that is similar to bone [5]. Furthermore, Mg’s conductivity makes it promising for neural implant applications [6], [7], since studies have shown that the conductive properties of neural implants play a key role in supporting neuronal growth and reducing glial scar tissue formation [8]. As a biodegradable implant material, Mg eliminates the necessity of secondary surgeries for implant removal. Moreover, Mg ions, one of the degradation products of Mg, alleviate pathological conditions associated with imbalance of Mg ion levels [9]. Clinically, Mg sulfate solution has been administered intravenously for treating aneurysmal subarachnoid hemorrhage and eclampsia [10], [11]. In short, Mg-based metals can provide biomedical implants and scaffolds with beneficial properties for improved clinical outcomes.

One of the main challenges in using Mg-based biomaterials is its rapid degradation, which causes adverse effects on the local physiological environment due to high Mg ion concentrations, alkaline pH conditions, and release of hydrogen gas. Mg degrades by reacting with water through the following overall reaction:
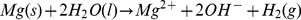
(1)


Previous studies have shown that degradation of Mg was initially rapid as indicated by acute pH increase during the first 24 hours, but slowed down after 24 hours because a degradation layer forms on the surface [12], [13]. Therefore, to compare with polished metallic Mg, Mg samples that were pre-degraded in the cell culture for 24 hours were investigated as a possible means to alleviate the effects induced by initial acute degradation.

Literature reports on the cytocompatibility of Mg-based materials are inconsistent due to lack of standardized protocols [14]. Because the cell types, material processing parameters, and sample surface preparation procedures vary, it is difficult to directly compare the results of these studies [5], [13], [15], [16]. Furthermore, studies in current literature did not distinguish the role of each factor among all contributing factors (e.g. Mg alloy design and processing, elevated Mg ion concentrations, and increased pH) on the observed cell responses. Therefore, we developed an *in vitro* model to investigate the combined and individual factors of Mg degradation on cell behavior in this study. The knowledge on the cellular functions in response to the respective Mg degradation products (i.e., hydroxide ions and Mg ions) will provide a useful design guideline for Mg-based implants/scaffolds prior to *in vivo* studies and clinical translation.

We attempted the use of human embryonic stem cells (hESCs) as our screening model due to their high sensitivity to chemicals and toxicants, their capability of prolonged proliferation, and their ability to differentiate into three germ layers for regenerative medicine [17]. ESCs provide a sensitive *in vitro* model to assess the possible adverse effect of Mg alloys in the early stages of the implant or scaffold development to minimize the potential risk in animal and clinical studies later. The validated embryonic stem cell test (EST) utilizes mouse embryonic stem cells as a predictor for embryo toxic compounds because embryos and fetuses are more sensitive to environmental toxicants than adults are [18]. However, this mouse-based EST often does not faithfully correlate to cellular responses in human [19]. Thus, to eliminate the concerns on the differences of species, the human ESC model was used for this *in vitro* study. Moreover, previous studies confirmed the greater sensitivity of hESC model in detecting subtle cytotoxic effects [20], [21]. Specifically, Cipriano et al. investigated the effects of four different Mg-Zn-Sr alloy compositions on their degradation properties and determined their cytocompatibility using the hESC model [20], [21]. Although Cipriano et al. demonstrated the efficacy and sensitivity of the hESC model in screening Mg alloys, the exact mechanisms of observed hESC death remained elusive. In an effort to elucidate the possible causes for hESC death, we not only compared the *in vitro* hESC responses to pure Mg and pre-degraded Mg, but also extensively investigated the combined and individual effects of Mg degradation products released to hESC culture. For the purpose of mechanistic studies, commercially pure Mg was selected to eliminate additional variables induced by different alloying compositions and processing parameters. Preliminary results showed that induction of pure Mg samples to hESC culture led to cell death after 30 hours of culture [22]. In this study, we further investigated the proliferation and pluripotency of hESCs in response to Mg and its degradation products to reveal underlying mechanisms and to understand the respective effects of pH and Mg ions on hESC behavior.

Last but not the least, hESCs provide an attractive therapeutic option for cell replacement therapy and regenerative medicine considering their ability to proliferate and differentiate into different cell types, such as neurons, osteoblasts, or fibroblasts, for the targeted tissue regeneration. The effects of Mg ions of various concentrations on the pluripotency of hESCs were examined for the first time in this study to determine the feasibility of Mg-based biodegradable scaffolds for maintaining hESC pluripotency for potential stem cell therapies. Rather than isolating relevant adult cells from animals or human tissues that have limited expansion capacity, hESCs have greater capacity for proliferation, thus reducing the number of animals and donors needed for tissue and cell harvests. Moreover, hESC model provides an *in vitro* system for studying genetic and degenerative diseases; and this disease-in-a-dish model is useful for understanding cellular responses to Mg-based implants or scaffolds in patients with specific diseases. Additionally, magnesium sulfate (MgSO_4_) is used clinically to treat pregnant women with eclampsia, but its safety and mechanism are still unknown [23]; and this study elucidates the possible embryo toxicity of elevated Mg ion concentrations.

## Materials and Methods

### Preparation and Characterization of Mg Samples

As-rolled 99.9% pure Mg sheets (Good Fellow, Coraopolis, PA) were grinded using 600, 800, and 1200 grit silicon carbide abrasive papers (PACE Technologies, Tucson, AZ) to remove the oxide layer and expose the metallic Mg on the surface. The Mg with the metallic surface was referred to as M-Mg in this study. The M-Mg sheets had a thickness of 250 µm and were cut into 5×5 mm squares for surface characterization and hESC culture. Mg samples were individually weighed before the stem cell culture. The M-Mg surface was then disinfected with 100% ethyl alcohol (VWR, Radnor, PA) followed by ultraviolet (UV) radiation in a class II biosafety cabinet for 12 hours on each side. The surface microstructure and elemental composition of the M-Mg before cell culture were characterized previously using an XL30 FEG scanning electron microscope (SEM, Phillips, North Billerica, MA) with the attached detector for energy-dispersive X-ray spectroscopy (EDS; EDAX, Mahwah, NJ) [22].

### Human Embryonic Stem Cell Culture Experiments

#### 1. Preparation of the hESCs and culture media

H9 hESCs (WiCell, Madison, WI) were stably transfected with an OCT4-GFP reporter plasmid as previously described [24]. H9-OCT4 hESCs were maintained with mTeSR®1 media (STEMCELL Technologies, Vancouver, BC, V5Z 1B3, Canada) in T-25 flasks coated with Geltrex® (Invitrogen, Grand Island, NY) and Dulbecco’s Modified Eagle Medium (DMEM) (Invitrogen 11965092) (1∶50).

A solution of alkaline mTeSR®1 media was prepared to study the effects of alkaline pH condition on hESC behaviors. Specifically, mTeSR®1 media was adjusted to pH 8.1 using 0.1 M NaOH, as this pH 8.1 was the average pH of media cultured with the Mg samples for 24 hours.

A 4 M of Mg ion stock solution was prepared by dissolving 0.837 g of MgCl_2_•6H_2_O into 1 mL DI water and sterilized by filtering through a 0.20 micron filter (Sartorius Stedim, Bohemia, NY). This 4 M of Mg ion stock solution was sterilely mixed with mTeSR®1 media in a ratio of 1∶100, 3∶400, 1∶200, 1∶400, 1∶1000, and 1∶10000 to obtain the mTeSR®1 media supplemented with 40, 30, 20, 10, 4, and 0.4 mM Mg ions, respectively. The baseline Mg ion concentration in mTeSR®1 media was 0.56 mM [25].

#### 2. Preparation and characterization of pre-degraded Mg samples

Upon reaching 80–90% confluency, the H9-OCT4 hESCs were passaged and split at a ratio of 1∶5. Accutase (Innovative Cell Technologies, San Diego, CA), and glass beads were used to detach the cells from the flask surface. These detached cells were then centrifuged at 800 rpm for 3 minutes, resuspended in mTeSR®1 media and incubated in the Geltrex®-coated wells of a tissue-culture-treated 12-well plate under standard cell culture conditions (that is, a sterile, 37°C with 5% CO_2_/95% air, humidified environment). After 24 hours of incubation for cell attachment, the mTeSR®1 media were replenished, and four M-Mg samples were placed in the transwell® inserts (Corning, Union City, CA) and inserted into the wells where H9-OCT4 hESCs were cultured. After 24 hours of incubation, the Mg samples were collected, dried in a vacuum oven at room temperature for 24 hours, and weighed under sterile conditions. These samples were pre-degraded in the cell culture and referred to as D-Mg. The pH and Mg ion concentration of the post-culture media were measured, and these values were used as the basis for selecting the alkaline pH conditions and supplemental Mg ion dosages.

The surface microstructure and elemental composition of the D-Mg sample were characterized by SEM and EDS respectively, at an accelerating voltage of 15 kV. The insoluble precipitates and degradation products were kept intact on the surface to gain a better understanding of the biodegradation process. EDS analysis was performed at a magnification of 2000X so that a substantial portion of the sample surface would be analyzed.

#### 3. The hESC culture with the Mg samples and the experimental media conditions

Three 12-well plates were prepared similarly as described above for *in vitro* stem cell experiments with the M-Mg and D-Mg samples, alkaline pH condition, and supplemental Mg ion dosages. Briefly, H9-OCT4 hESCs were seeded into the Geltrex®-coated wells with 1 mL of resuspended cells and incubated under standard cell culture conditions for 24 hours. For plate one, mTeSR®1 media were replenished and the M-Mg and D-Mg samples were introduced through transwell® inserts after 24 hours of incubation. For plate two, the mTeSR®1 media were removed and replaced with the alkaline mTesR®1 after 24 hours. For plate three, the media were replaced with the mTeSR®1 media supplemented with 0.4, 4, 10, 20, 30, 40 mM Mg ion solutions. In each of these plates, the wells without any Mg samples or the experimental media conditions were included as the control. These three plates were placed into an incubator with a live cell imaging system (Nikon BioStation CT, Melville, NY) and maintained under standard cell culture conditions. Two points in each well were set for time-lapse phase contrast and fluorescence images every 6 hours for 72 hours. After the images were taken at 24, 48, and 72 hours, the post-culture media were collected for analysis. The media were replenished every 24 hours with the corresponding fresh mTeSR®1 media, alkaline mTeSR®1 media, or Mg ion supplemented mTeSR®1 media. All experimental conditions were carried out in triplicate.

### Sample Characterization and Media Analysis after Cell Culture

Mg samples were taken out from the media after 72 hours of culture, dried at room temperature in vacuum, and weighed for final mass. The pH of the media collected at 24, 48, and 72 hours from all experimental conditions was measured using a pre-calibrated pH meter (VWR, Model SB70P, Radnor, PA). Mg ion concentrations in the collected post-culture media were determined using inductively coupled plasma - atomic emission spectroscopy (ICP-AES; Perkin Elmer Inc., Optima 2000 DV, Wellesley, MA). A standard curve was generated by running the solutions containing 250, 125, 62.5, 31.25, and 15.63 mg/L (10.29, 5.14, 2.57, 1.29, 0.64 mM) of MgCl2·6H2O. The samples were diluted with deionized water accordingly to ensure the measured values were within the range of the standard curve. The ICP-AES measurements of the samples and the standards were all carried out on the same day under the same conditions.

### Immunocytochemistry and Fluorescence Imaging

After 72 hours of culture, the hESCs were washed three times with 1X phosphate buffered saline (PBS) and fixed with 4% paraformaldehyde for 10 minutes. After fixation, the cells were washed 3 times with PBS, and blocked for 1 hour with an antibody staining solution consists of PBS, 1% donkey serum (Sigma, St. Louis, MO), and 0.1% Triton X-100 (Sigma). The cells were incubated with the following antibodies overnight at 4°C: anti-Sox2 (1∶200, R&D systems, Minneapolis, MN), anti-SSEA3 (1∶40, Millipore, Billerica, MA), and anti-OCT4 (1∶200, Santa Cruz Biotechnology, Dallas, TX). These cells were then washed 3 times with PBS, incubated with Alexa Fluor® 488 or Alexa Fluor® 594 secondary antibodies (Life Technologies) at room temperature for one hour, and mounted in the VECTASHIELD® mounting medium with DAPI (Vector Laboratories, Burlingame, CA). Fluorescence images were taken using an inverted fluorescence microscope (Nikon, Eclipse T*i*, Melville, NY).

### Cell Proliferation Analysis

Fluorescence images from immunostaining were used to quantify the number of cells expressing pluripotency markers at the endpoint of 72-hour culture. Specifically, SOX2 and DAPI stained cells were counted in six randomly selected regions using ImageJ software to calculate the average cell density (cells per unit area) and standard deviation. The time-lapse phase contrast and fluorescence images recorded by the Biostation CT were used to quantify the coverage area of viable H9-OCT4 hESC colonies. ImageJ software was used to manually outline the coverage area of viable cell colonies expressing GFP driven by the OCT4 promoter. The percentage of area covered by viable cell colonies was calculated using the outlined area divided by the total image area, multiplied by 100. Viable cell colonies were quantified based on phase contrast images because the tightly packed colonies of hESCs had well-defined external boundaries and morphologies, and the hESCs had a high nucleus to cytoplasm ratio [26].

Since each experimental condition was tested in triplicate wells with the time-lapse images at two different areas in each well, 6 images were analyzed and averaged for each test condition. Furthermore, the coverage area of viable cell colonies was normalized over the first recorded time point for each respective condition. These image based methods were used for cell viability and proliferation studies instead of the commonly used tetrazolium based assays (e.g. MTT assay) because Mg ions converts tetrazolium salts to formazan and thus interferes with the results [27]. That is, MTT assay yields greater cell viability values than the actual in the presence of Mg ions, which is misleading [27].

### RNA Extraction and Analysis with Quantitative Polymerase Chain Reaction

H9-OCT4 cells were collected at the endpoint of 72-hour culture. The total RNA was extracted for each group of cells using the ZR RNA MicroPrep Kit (Zymo Research, R1061, Irvine, CA). The RNA concentrations were quantified using a multi-volume multi-sample spectrophotometer system with Gen5 software (BioTek Epoch™, Winooski, VT). RNA from the sample extraction was reverse-transcribed with iScript cDNA synthesis kit (BioRad, Hercules, CA) on a thermal cycler (BioRad, MyCycler). cDNA was subjected to the real-time quantitative polymerase chain reaction (qPCR) analysis using TaqMan probes (Life Technologies) on a CFX384 instrument (BioRad, CFX384). The gene-specific probes tested and housekeeping genes were listed in Table 1. Housekeeping genes were used as endogenous controls to normalize gene expression. The qPCR cycling temperatures and times were set initially at 95°C for 5 min, followed by 40 cycles at 95°C for 1 min, 60°C for 1 min, and 72°C for 1 min, and the last extension step was set at 72°C for 5 minutes. Each reaction was performed in triplicate.

**Table 1:** Genes screened by Q-PCR to determine pluripotency of hESCs.

Gene Type	Genes
Housekeeping	18S, ACTB, GAPDH
Human ES	POU51 (OCT4), SOX2, NANOG, KLF4,ZFP42 (REX1), cMYC
Endoderm	SOX17, PRDM1 (BLIMP1)
Mesoderm	GOOSECOID
Neuroectoderm	PAX6

### Statistical Analysis

A script was written in the program R for the Shapiro-Wilk normality test, the F test, and the standard analysis of variance (ANOVA) test. Standard *post-hoc* analysis with the Holm-Bonferroni correction was used on the ANOVA test results. Values of *p*<0.05 for the Shapiro-Wilk test and F tests were used to verify the normality and the variance of the data. Values of *p*<0.05 were considered statistically significant.

## Results

### Surface Characterization of the Mg Samples and the hESC Responses

**Figure 1:**
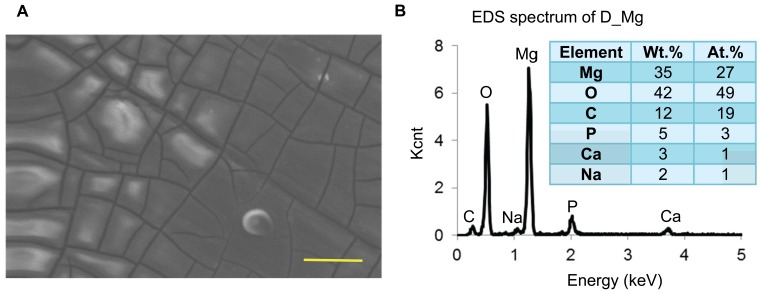
Surface morphology and composition of D-Mg. (A) SEM image and (B) EDS analysis of the D-Mg sample, showing the presence of a degradation layer on the surface. Accelerating voltage: 15 kV. Original Magnification: 2000x. Scale bar = 20 µm.

The scanning electron microscopic (SEM) image and energy dispersive X-ray spectroscopy (EDS) analysis of the D-Mg surface in Figure 1 confirmed the presence of a degradation layer on the pre-degraded Mg samples. While M-Mg surface has previously been shown to be smooth with traces of polishing marks and a composition that mainly consists of Mg [22], the D-Mg surface in this present study showed corrosion cracks around the grain boundaries and a composition that consisted of 27 at.% Mg, 50 at.% oxygen (O), 19 at.% carbon (C), 3 at.% phosphorous (P), 1 at.% calcium (Ca), and 1 at.% sodium (Na). These components of the degradation layer after 24 hours of culture with hESCs likely resulted from the degradation products of Mg, precipitation of inorganic salts from the culture media, and the interactions among Mg, its degradation products, and the media components [13].

Previous study on Mg cultured with mesenchymal stem cells in Dulbecco's Modified Eagle Medium (DMEM) for 24 hours showed the presence of similar elements (Mg, O, P, Ca, and Na), with the exception of C on the surface [12]. In this study, we detected the presence of C on the surface, possibly from the proteins and lipids in the mTeSR®1 media [25]. The ionic compositions of the mTeSR®1 media and DMEM were listed in Table 2 in comparison with human blood plasma [25], [28]. Although we speculated that the media composition and ionic strength might affect Mg degradation process, rate, and composition of the degradation layer on the surface [28], the exact mechanism is still uncertain. The ionic strength of the mTeSR®1 media used in this study were less than that of DMEM and human blood plasma, as shown in Table 2. Human blood plasma represents the typical physiological environment that Mg-based implants or scaffolds are exposed to in the human body.

**Table 2:** The ion concentrations of inorganic salts in the culture media in comparison with human blood plasma [25], [28].

	Concentration (mM)		
Ions	mTeSR®1	DMEM	Human Blood Plasma
**Na^+^**	113.74	155.31	142
**K^+^**	3.26	5.33	5
**Li^+^**	0.98	–	–
**Mg^2+^**	0.56	0.81	1.5
**Ca^2+^**	0.82	1.80	2.5
**Fe^3+^**	–	0.0002	–
**Cl^−^**	100.96	119.27	103
**HCO_3_^−^**	18.00	44.05	27
**NO_3_^−^**	–	0.0007	–
**H_2_PO_4_^−^**	0.36	0.92	–
**HPO_4_^2−^**	0.39	–	1
**SO_4_^2−^**	0.32	0.81	0.5
**Ionic Strength**	0.12	0.17	0.15

**Figure 2:**
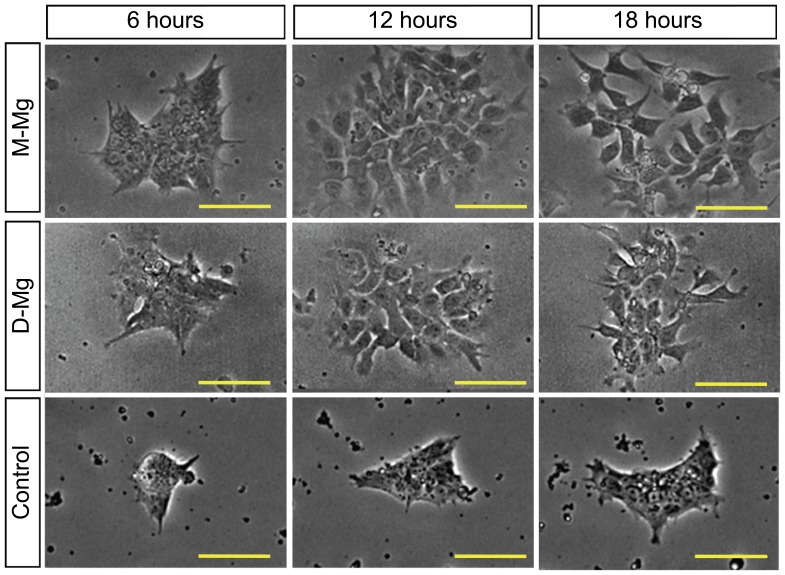
Phase contrast optical images of hESC colonies cultured with M-Mg and D-Mg. As compared with the control, the H9-OCT4 hESCs exposed to the M-Mg and D-Mg showed increased dispersion at 12 and 18 hours of culture, and thus greater coverage area of cell colonies. Scale bar = 100 µm.

A preliminary study on the cytocompatibility of M-Mg and D-Mg samples showed rapid hESC death within the first 30 hours of culture [22]. A closer look at the phase contrast images of hESCs revealed that the hESCs cultured with the M-Mg and D-Mg showed a more dispersed morphology as compared with the blank control without Mg samples at 12 and 18 hours, as shown in Figure 2. This change of colony morphology correlated with the initial increase of coverage area of viable hESC colonies at 12 and 18 hours as reported in the preliminary study [22].

**Figure 3:**
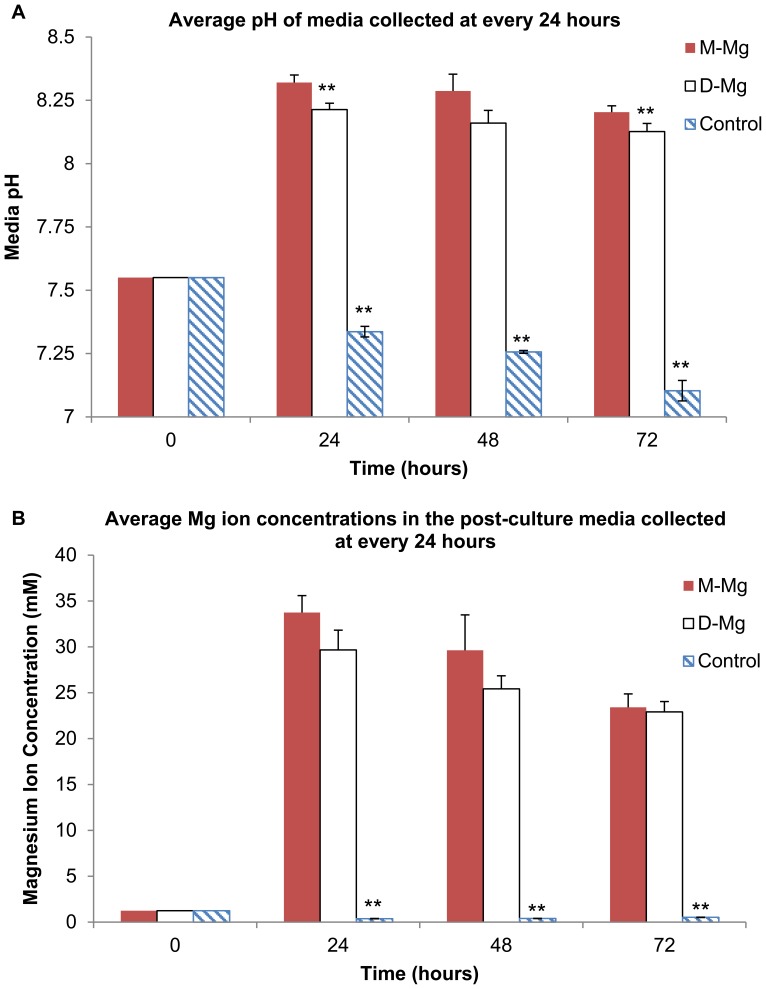
Media pH and Mg ion concentrations in the media collected at every 24 hours after hESCs were cultured with M-Mg and D-Mg for 72 hours. (A) The average pH and (B) the average magnesium ion concentrations (**p<0.05 compared to M-Mg). Values are mean ± standard deviation: n = 3. The average pH and Mg ion concentrations for the culture with both Mg samples significantly increased as compared with the control without Mg samples. The statistically significant lower pH of the post-culture media with D-Mg was detected in comparison with M-Mg. Furthermore, after 24 hours, a general trend of gradual decrease in average pH and magnesium ion concentrations over time was observed for both Mg samples. (**p<0.05 compared to M-Mg). Values are mean ± standard deviation: n = 3.

The results of the post-culture media analysis in Figure 3 showed that the media pH and Mg ion concentrations for the culture with both Mg samples significantly increased as compared with the control without Mg samples. This increase in media alkalinity and Mg ion concentrations was expected as a result of Mg degradation as described in Reaction 1 in the Introduction. Specifically, the media pH increased from the initial value of 7.55 to a range of values from 8.1 to 8.3 as shown in Figure 3A, while the average Mg ion concentrations increased from 1.2 mM to values that ranged from 23 mM to 33 mM as shown in Figure 3B. A general trend of gradual decrease in average pH and Mg ion concentrations over time was observed for both Mg samples. A statistically significant difference in post-culture media pH was detected between the M-Mg and D-Mg samples at 24 and 72 hours, indicating slightly lower pH for the media cultured with D-Mg. No statistically significant difference in Mg ion concentrations was detected between the M-Mg and D-Mg samples at all the time points. In addition, the mass measurements showed that the percentages of average mass loss for the M-Mg and D-Mg samples at the end of 72 hours of culture were 24% and 23%, respectively, with no statistically significant difference.

### The hESC Response to the Alkaline Media Condition

**Figure 4:**
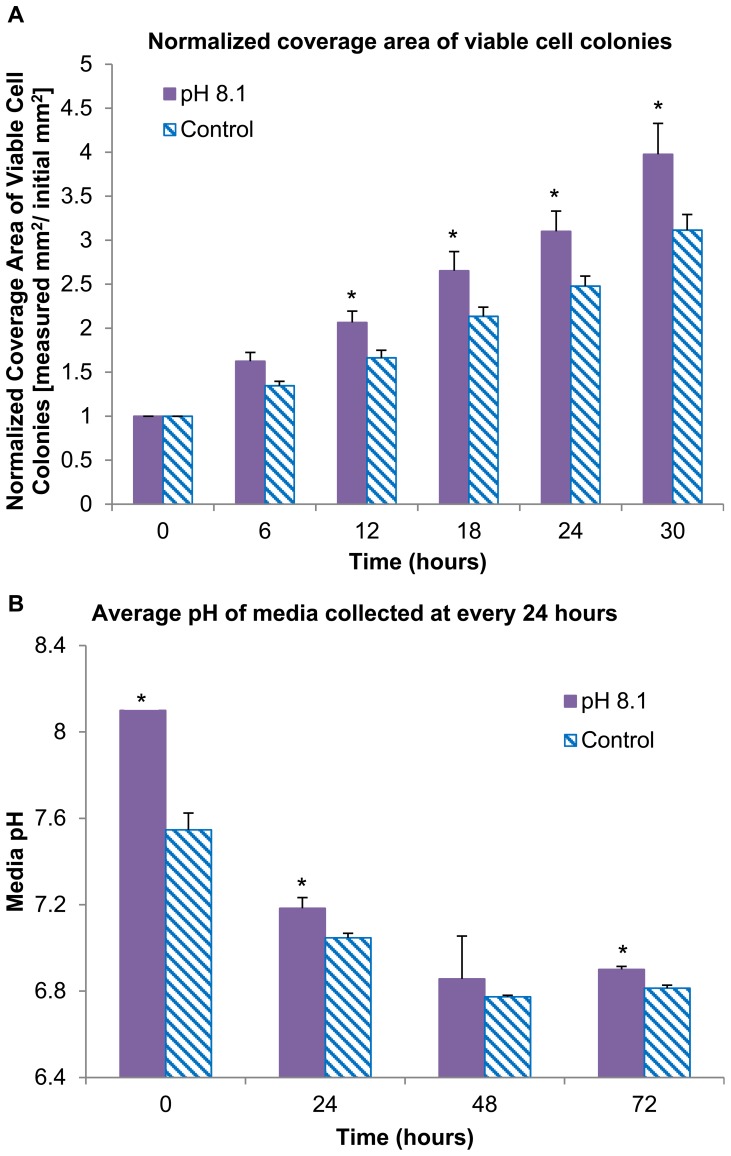
Coverage area of viable hESC colonies and pH of post-culture media under the alkaline or normal media conditions. (A) The coverage area of viable hESC colonies at 6–30 hours of culture after being normalized over the first time point at 6 hours. Values are mean ± standard error of the mean: n = 6. The hESCs cultured under the alkaline media condition proliferated and grew to confluency, indicating that the initial increase of media pH to 8.1 was not the direct cause of cell death observed in the cultures with the Mg samples. (B) The average pH of post-culture media collected at every 24 hours for 72 hours (*p<0.05 compared to the control). Values are mean ± standard deviation: n = 3.

The specific effect of alkaline media on the hESC response was investigated because the elevated media pH was caused by hydroxide ions, one of the degradation products of Mg samples. The pH of mTeSR®1 media was specifically adjusted to 8.1 before pipetting into the culture wells, because this pH value represented the average media pH when the polished metallic Mg was pre-degraded in hESC cell culture for 24 hours. The H9-OCT4 hESCs cultured with the alkaline media still proliferated and grew into confluency, with the normal undifferentiated morphology similar to that of the control wells throughout the 72 hours of culture (image not shown). Moreover, as shown in Figure 4A, the normalized coverage area of viable cell colonies increased under alkaline media condition as compared to the control at each time point of 6–30 hours, with statistically significant differences (*p*<0.05) detected. Specifically, after 30 hours of culture, the viable colony coverage quadrupled under alkaline media conditions, and only tripled for the control, as compared with the initial value at time zero. This greater coverage area of viable cell colonies under the alkaline media condition indicated that the initial increase of media pH did not cause adverse effects on hESC proliferation. Therefore, the initial increase of media pH to 8.1 might not be the direct cause of cell death observed in the cultures with the Mg samples.

Figure 4B shows that the post-culture media had higher pH values for all of the time points under alkaline media condition as compared with the control, with statistically significant difference detected at time zero, 24 and 72 hours. Furthermore, the pH of all the post-culture media decreased over time and dropped to ∼7 after 48 hours, possibly due to the regular metabolic processes of the hESCs that released acidic metabolites.

### The hESC Response to the Supplemental Mg Ion Dosages

**Figure 5:**
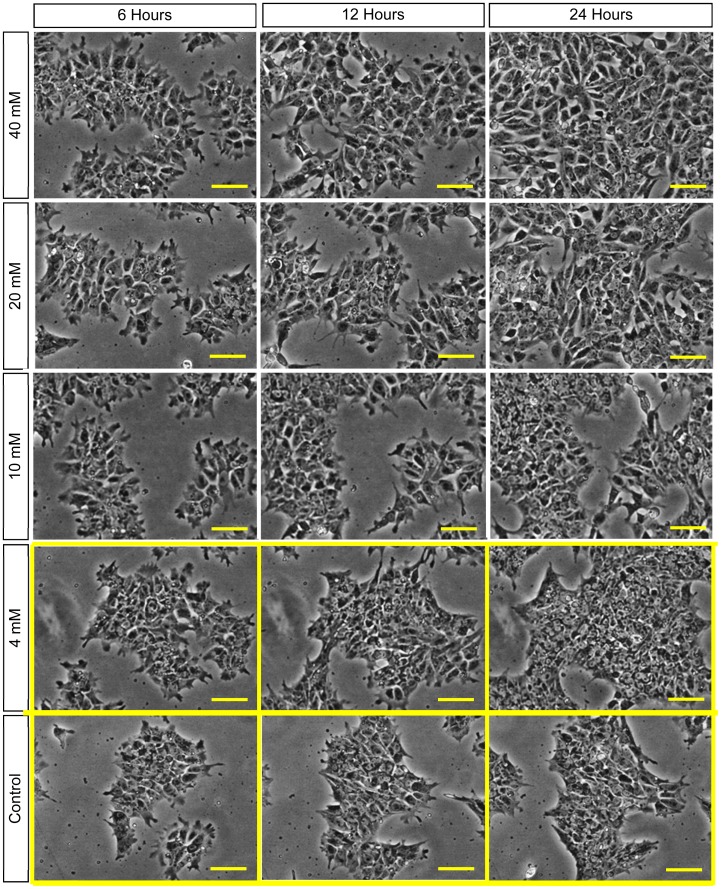
Phase contrast optical images of hESCs cultured in the media supplemented with Mg ion dosages. The hESC colonies showed a tightly packed morphology in the media supplemented with 4 = 100 µm.

The specific hESC response to excess Mg ions in the media was investigated because Mg ion was one of the major degradation products released during Mg degradation. The H9-OCT4 hESCs proliferated and grew to confluency in the mTeSR®1 media supplemented with 0.4–40 mM of Mg ion dosages, as shown in Figure 5. The time-lapse images revealed that the hESCs maintained their tightly packed colony morphologies throughout the culture period in the media supplemented with 0.4–4 mM Mg ions, similar to the control wells, as shown in Figure 5. The images for 0.4 mM media condition were not shown as they were very similar to the control. When the supplemental dosages of Mg ions increased to a range of 10–40 mM, however, the hESCs lost their tightly packed morphology, with a decrease in cell-to-cell adhesion, as shown in Figure 5. Evidently, the hESCs became more dispersed with greater cellular extensions, and thus appeared to have greater coverage area at the Mg ion dosages of 10 mM and greater.

**Figure 6:**
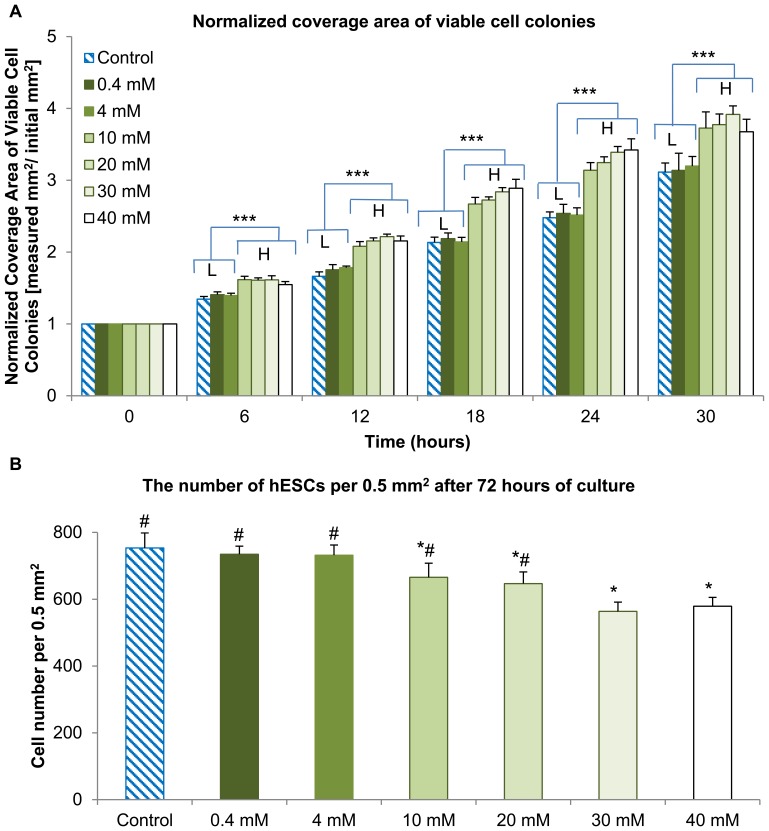
Coverage area of viable hESC colonies and the numbers of hESCs when cultured in the media supplemented with Mg ion dosages. (A) The coverage area of viable hESC colonies after being normalized over the first time point at 0 hour in the cultures with respective supplemental Mg ion dosages (***p<0.05 when comparing L-group with the H-group). (B) The average numbers of hESCs per 0.5 mm^2^ after being cultured for 72 hours and immunostained with DAPI and pluripotency marker-SOX2 (*p<0.05 compared to the control and *#p*<0.05 compared to 40 mM). Although the normalized coverage area of viable hESC colonies was greater at supplemental Mg ion dosages of 10 mM and greater, the cell counts per unit area were actually lower. This confirmed that the supplemental Mg ion dosages of 10 mM and greater caused cell dispersion and loss of tightly packed morphology. Values are mean ± standard error of the mean: n = 6.

The analysis on the coverage area of viable cell colonies, as shown in Figure 6A, revealed two distinct groups: one group composed of the control, 0.4 and 4 mM that showed lower coverage area (referred to as L-group), and the other group of 10, 20, 30, 40 mM that showed higher coverage area(referred to as H-group). The normalized coverage area of viable hESC colonies showed no statistically significant differences (*p>*0.05) within the L-group or within the H-group at every time point. The normalized coverage area of viable hESC colonies in the H-group were greater than the control, with statistically significant differences (*p*<0.05) at all the time points. The increased coverage areas of viable hESC colonies were most likely due to the changes of hESCs from tightly packed to loosely dispersed morphologies in the media supplemented with Mg ion dosages above 10 mM, without an actual increase in the cell number as indicated in Figure 6B. In other words, at the end of 72 hours of culture, the average number of hESCs per 0.5 mm^2^ decreased as the supplemental Mg dosages increased, as shown in Figure 6B. Generally, all the DAPI-stained cells expressed the pluripotency marker SOX2 as verified by overlaying the fluorescence images of DAPI and SOX2 stains. The cultures supplemented with 0.4 and 4 mM Mg ions showed similar cell counts as the control. In contrast, the cultures supplemented with 10–40 mM Mg ions showed less cell counts than the control; and the statistically significant difference (*p*<0.05) was detected.

**Figure 7:**
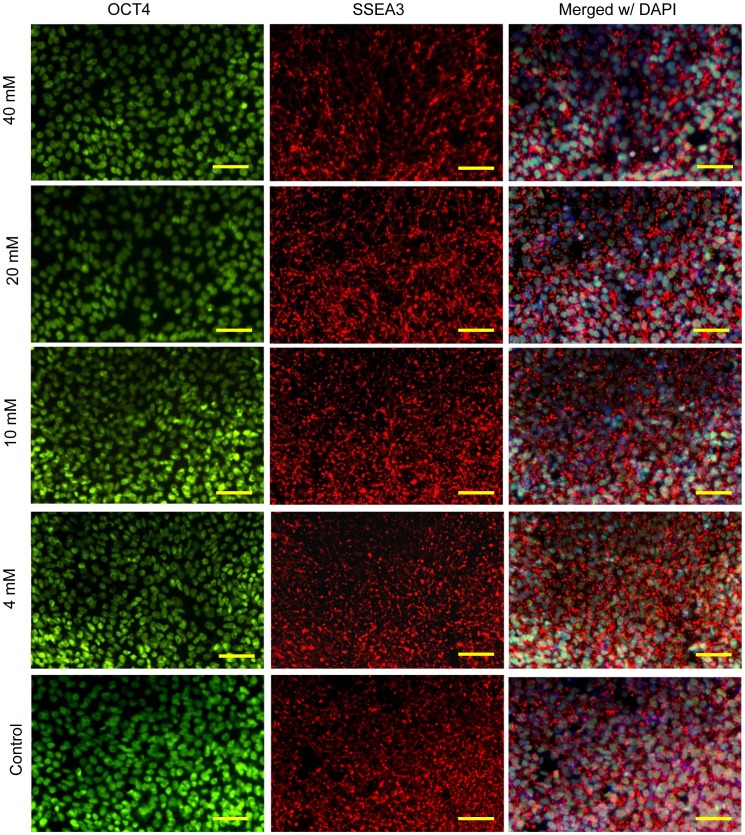
Media pH and Mg ion concentrations in the post-culture media collected at every 24 hours after hESCs were cultured in the media supplemented with Mg ion dosages. (A) Average pH and (B) Mg ion concentrations in the post-culture media when the supplemental Mg ion dosages ranged from 0.4–40 mM (*p<0.05 compared to the control at each time point respectively). Values are mean ± standard deviation; n = 3. The cell counts in the hESC cultures supplemented with 30 and 40 mM Mg ions were statistically lower than the cultures with lower Mg ion dosages and the control, and, therefore, less acidic metabolites formed and the pH values were statistically higher. Additionally, statistically greater Mg ion concentrations were observed in the post-culture media at the critical Mg ion concentration of 10 mM and above, as compared with the control, 0.4 mM and 4 mM media conditions at each respective time point.

Figure 7A shows the pH values of the post-culture media at every 24 hours over a 72-hour period. For all the test conditions and the control, the pH values decreased at the 48 and 72 hours as compared with that at 0 and 24 hours. This increased acidity at the later stages possibly resulted from the release of acidic metabolites into the media during cell proliferation. No statically significant difference was detected among all the test conditions and the control at the 24 hours. At 48 and 72 hours, the media supplemented with 0.4–20 mM Mg ions maintained a pH comparable to that of the control (with the exception of the media supplemented with 4 mM Mg ions at 48 hours). In contrast, the media supplemented with 30 mM and 40 mM Mg ions had significantly higher pH values when compared with the control at 48 and 72 hours, respectively.

Figure 7B shows the Mg ion concentrations in the post-culture media as measured by the ICP-AES. Statistically significant differences were detected when the supplemental Mg ion dosages increased to 10 mM and greater, as compared with the control, 0.4 mM and 4 mM media conditions. As the supplemental Mg ion dosage increased in the initial culture media, more Mg ions were consumed during culture. For example, after culturing hESCs for 24 hours in the media supplemented with 10 mM Mg ions, about 7–8 mM of Mg ions were left in the post-culture media. After culturing hESCs for 24 hours in the media supplemented with 40 mM Mg ions, approximately 24–25 mM of Mg ions were left in the post-culture media.

**Figure 8:**
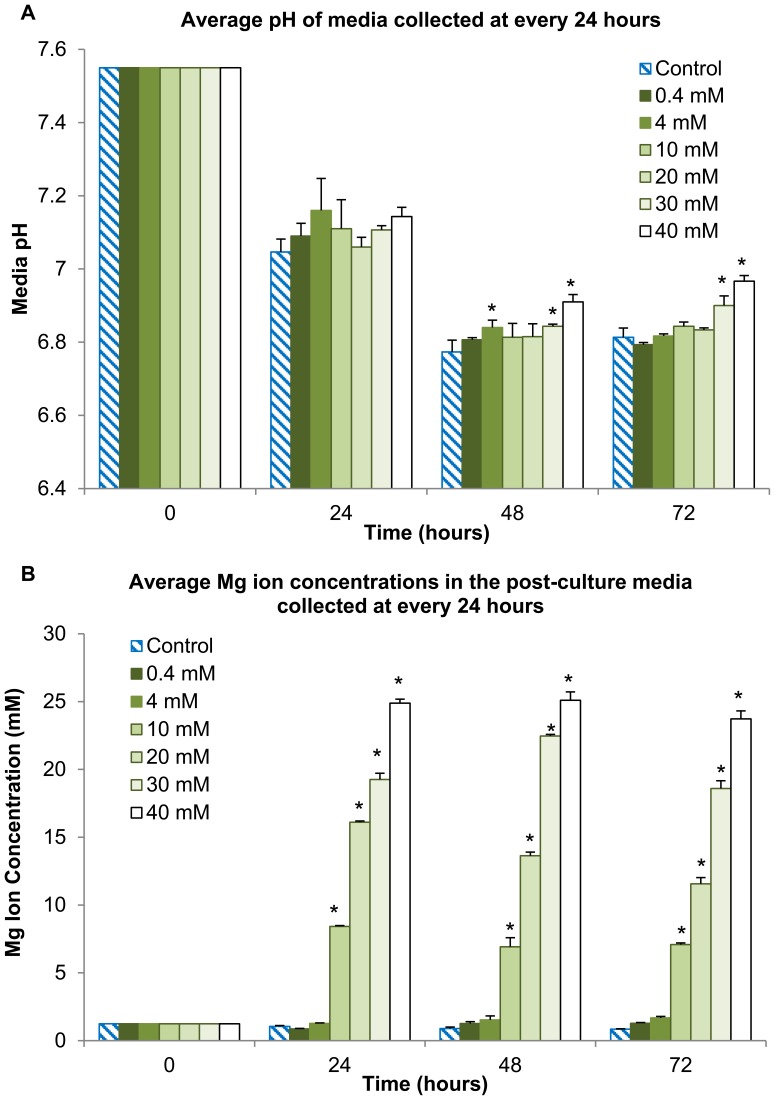
Immunocytochemistry analysis on the hESC expression of OCT4 and SSEA3 after being cultured in the media supplemented with Mg ion dosages. All H9-OCT4 hESCs cultured in the media supplemented with different dosages of Mg ions expressed markers for OCT4 and SSEA3. The images for 0.4 mM media condition were not shown as they were very similar to the control. Nuclear localization of OCT4 is shown in green (left column) and SSEA3 expression on the cell surface is shown in red (middle column). The right column shows merged images of the OCT4, SSEA3, and DAPI (blue, cell nucleus). Scale bars = 100 µm.

**Figure 9:**
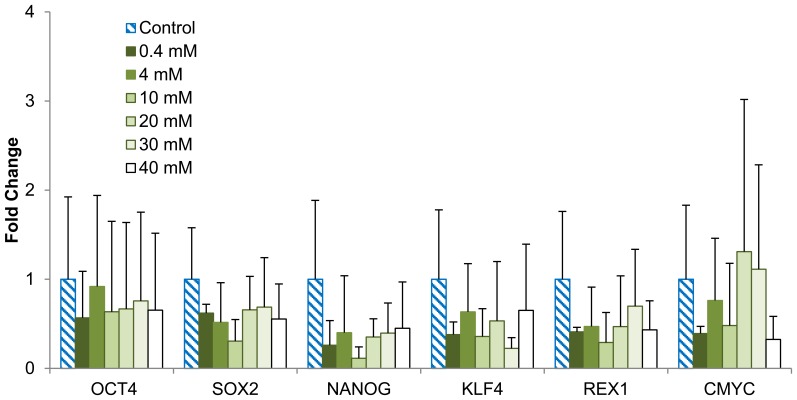
Quantitative PCR analysis of pluripotency markers for hESCs after being cultured in the media supplemented with Mg ion dosages. H9-OCT4 hESCs were cultured for 72 hours and analyzed for expression of the corresponding markers. No statistically significant difference was detected among any of the groups. Values are mean ± standard deviation; n = 9.

In Figure 8, the immunocytochemistry analysis on the pluripotency of H9-OCT4 hESCs revealed the expression of OCT4 and SSEA3 on the hESCs cultured in the media supplemented with 4–40 mM Mg ions for 72 hours. The images for 0.4 mM media condition were not shown as they were very similar to the control. Real-time qPCR analysis showed that the expressions of several pluripotency-related genes – OCT4, SOX2, NANOG, KLF, REX1, and cMYC – reduced slightly in the culture supplemented with 40 mM Mg ions, as shown in Figure 9. However, no statistically significant difference was detected among all the testing groups due to the large data deviation. We also assessed the expressions of early differentiation markers in the cells for potential lineages, including SOX17 (endoderm), GOOSECOID (mesoderm), PAX6 (neuroectoderm) and BLIMP1 (germ cell). The hESCs cultured in the media with the supplemental Mg ion dosages did not appear to express any of the differentiation markers after 72 hours of culture.

## Discussion

The objective of this study was to investigate the effects of Mg and its degradation products (Mg ions and hydroxide ions) on the viability, proliferation, and pluripotency of hESCs. Test conditions of the alkaline media and the supplemental Mg ion dosages were selected based on the measured pH values and Mg ion concentrations when M-Mg was pre-degraded for 24 hours. Since it was previously shown that the Mg samples induced cell death within the first 30 hours of culture [22], we designed additional testing conditions to determine the potential causes of the cell death. The alkaline media condition was set to be at the pH of 8.1, similar to the pH of media containing the metallic Mg, while the supplemental Mg ion dosages were tested at a range of 0.4 mM to 40 mM, which represented the range of normal physiological to therapeutic level. For example, the Mg ion dosage used for treating eclampsia ranges from 16–24 mM (4–6 g intravenously) [29], and this value is within the tested range of magnesium ion concentrations in this study. Using the sensitive hESC model, we are able to assess cytocompatibility of Mg-based implants or scaffolds, differentiate the specific effects of Mg ions and alkaline pH on the hESC behavior, and identify possible adverse effects of Mg at the early stages of development to minimize the potential risks in animal and clinical studies later.

### The Effects of Surface Conditions on Mg Degradation and Cytocompatibility

Various studies with Mg and its alloys have focused on surface modifications to delay Mg degradation, since surface conditions have been shown to play an important role in Mg degradation and cytocompatibility [12], [30], [31]. For example, in a study describing the effects of surface conditions (polished versus oxidized surfaces) of Mg-yttrium (Y) alloy on their interactions with bone marrow derived mesenchymal stem cells (MSCs), the samples with polished surfaces had superior cell adhesion due to their slower degradation (slower rate of mass loss and lower overall pH increase) [30].

We modified the surface of metallic Mg (M-Mg) with a degradation layer to form pre-degraded Mg (D-Mg) samples that slightly slowed down the degradation. Although we did not detect statistically significant differences between the M-Mg and D-Mg samples at each tested time point in terms of their mass loss and release of Mg ions, the average Mg ion concentration over time showed a decreasing trend for both samples. Furthermore, the D-Mg showed a statistically significant lower pH value in post-culture media at 24 and 72 hours, indicating slower release of hydroxide ions than the M-Mg samples. As the degradation layer on the surface of the D-Mg did not provide sufficient protection for the sample degradation and the degradation rate did not significantly decrease, the D-Mg samples still induced cell death within the first 30 hours of culture, similar to the M-Mg samples. At 12 and 18 hours of culture, we observed that the hESC colonies lost their typical tightly packed undifferentiated morphologies in the cultures with D-Mg or M-Mg samples, and the cells appeared more dispersed and elongated. In an attempt to identify the possible mechanisms that caused the rapid cell death and morphological change, we tested the pH values of the post-culture media and Mg ion concentrations to serve as a guideline for the design of each testing condition.

### Cytocompatibility of Mg

In this study, we observed rapid cell death, which disagreed with the results reported in the literature [5], [13]. Specifically, Li et al. reported that murine marrow cells in contact with Mg samples did not show any signs of morphology change or cellular lysis under microscopic observation [5]. Yun et al. reported that Mg samples did not have significant effects on the viability and proliferation of osteoblasts (ATCC HTB-96 U2OS Osteosarcoma from human female tibia) [13]. Although the purity of Mg samples was similar (99.9% pure Mg used in the study of Li et al. and this study, and 99.95% pure Mg used in the study of Yun et al.), the experimental parameters and cell culture methods reported by Yun et al. and Li et al. were different. Specifically, Li et al. used Mg samples with a size of 2×2×2 mm (surface area of 24 mm^2^), while Yun et al. exposed the top surface of a 6 mm diameter Mg cylinder(surface area of 28 mm^2^) [13]. In contrast, the surface area of Mg samples used in the cell culture of this study was much greater, that is, 55 mm^2^ (5×5×0.25 mm Mg sheets exposed in 3D). Another difference was the cell type used. The hESCs used in this study are more sensitive to degradation products than immortalized osteoblasts and murine marrow cells because hESCs are more susceptible to the changes in the media conditions than ordinary human adult cell lines and animal cells. Additionally, the sample preparation procedures (i.e., polishing, cleaning, and sterilization methods) and the culture media used were different, which may affect the cell responses as well. Specifically, Li et al. first abraded and cleaned Mg samples using 200# waterproof abrasive paper, ultrasonically cleaned for 10 min in distilled water, and then sterilized in an autoclave at 373 K for 60 min. Yun et al. polished the top surface of the mounted Mg samples using diamond slurry, ultrasonically cleaned in ethanol, and then blown dry with a stream of nitrogen. Li et al. cultured murine marrow cells and Mg samples in RPMI-1640 medium supplemented with 15% horse-serum. Yun et al. used McCoy's 5A medium supplemented with 5% fetal bovine serum (FBS) and ascorbic acid. It has been reported previously that the composition of culture media affected Mg degradation rate [32], which is very likely to influence the cell responses observed.

Clearly, inconsistencies on cytocompatibility studies of Mg-based materials are present across literature. In some studies, direct material exposure methods were utilized [5], [13], while in other studies media extract methods (also called indirect contact methods) were utilized [33], [34]. Specifically, in the direct material exposure method, the Mg samples were placed directly in the cell culture environment, while in the media extract method, the media extracts harvested from the sample degradation were added into the cell culture. We used the direct material exposure method in this study to expose the Mg samples to the hESC culture through transwell® inserts. Even within each type of methodology, there were still significant differences in experimental procedures. The differences in the sample preparation procedures, the sample surface area exposed, the cells used, and the culture media used, all affected the *in vitro* results and made it difficult to directly compare these results across literature. To address the problems induced by inconsistent methodology, we investigated the respective cytocompatibility effects of the key Mg degradation products, in addition to direct material exposure method.

### Effects of Alkaline Media pH on Proliferation and Morphology of hESCs

The lower-end value for the elevated pH range of post-culture media was 8.1, when the M-Mg and D-Mg samples were introduced into the cultures. Yun et al. also reported the same pH value of 8.1 in the osteoblast cell culture with Mg samples [13]. Therefore, we adjusted the culture media pH to 8.1 and investigated the effects of alkaline media on hESC viability and proliferation. The hESCs cultured under this alkaline media condition grew to confluency and did not seem to alter the cell and colony morphologies, indicating that this condition did not directly cause the hESC cell death observed in the M-Mg and D-Mg culture studies. In comparison, Grillo et al. reported that an immediate increase of pH to 8.6–9.1 following Mg particle immersion decreased UMR-106 rat osteosarcoma cell viability, suggesting pH of 8.6–9.1 might induce adverse effects on cell survival [35]. To further elucidate the exact effects of Mg degradation induced alkaline pH on cytocompatibility, we still need to continue to investigate the effects of more alkaline media conditions with a pH value greater than 8.1 and prolonged alkaline pH conditions on cell functions.

### Effects of Supplemental Mg Ions on Proliferation, Morphology, and Pluripotency of hESCs

In addition to the pH effect of Mg degradation, we examined the effect of Mg ions on hESC proliferation, morphology, and pluripotency. The hESCs cultured in the media with the tested supplemental Mg ion dosages of 0.4–40 mM grew to confluency, with a change in hESC morphology at dosages above 10 mM. The hESC morphology changed from tightly packed to a more dispersed geometries with elongated cellular extensions, and we observed decreased cell-to-cell adhesion. The hESCs cultured with the Mg samples showed very similar morphology change at 12 and 18 hours of culture, indicating that the elevated Mg ion concentrations may have been the contributing factor to changes in cell morphology. A morphology change in mouse embryo fibroblasts was also observed in previous studies, when these cells were cultured with the media supplemented with 250 mM MgSO_4_ [36].

The change in cell morphology with 10 mM or greater dosages of supplemental Mg ions correlated with the increase in normalized coverage area of viable cell colonies. This increase of hESC coverage area was more likely due to the morphology change as opposed to increased proliferation, because the supplemental Mg ion dosages above 10 mM also showed significantly lower cell numbers than the control at the end of 72-hour culture, as shown in Figure 6. This supplemental Mg ion dosage level of 10 mM can serve as a critical threshold value for the design and development of safe Mg based implants and scaffolds, at which adverse effects on the hESC functions started to appear. Feyerabend et al. performed cell viability studies on Mg salts using the MTT assay and showed that the critical Mg ion concentration was 10 mM [37]. However, another report showed that MTT assay was inappropriate for evaluating viability and proliferation of cells cultured with Mg-containing samples because Mg ions could also convert tetrazolium salts to formazan and skew the absorption spectra, thus producing false results [27]. Miki et al. used the Trypan blue exclusion test to determine viability of mouse embryo fibroblasts, and found that 250 mM was the critical Mg ion concentration at which a change of cell morphology and a decrease in cell viability (60%) were first observed [36]. This critical Mg ion concentration was significantly greater than our reported value of 10 mM, possibly because the hESC model used in our study was more sensitive than mouse cells.

The post-culture analysis of media pH and Mg ion concentrations in the Mg ion supplemented cultures supported the results observed on hESC proliferation. When compared with the control culture, a statistically significant difference in post-culture Mg ion concentrations was first observed at the critical Mg ion dosage of 10 mM. Furthermore, the post-culture media pH may also serve as an indicator for cellular metabolic activity, since a drop in the pH of the growth media typically indicates increased cellular metabolism and thus buildup of acidic metabolites. In one possible metabolic pathway, glucose is broken down into pyruvate via glycolysis, and pyruvate is converted into lactic acid via lactic acid fermentation. Since the cell counts in the hESC cultures supplemented with 30 and 40 mM Mg ions were statistically lower than that for lower Mg ion dosages as shown in Figure 6B, fewer cells produced less acidic metabolites, resulting in statistically higher pH values in cultures with higher Mg ion dosages as shown in Figure 7A.

Mg has the potential for tissue engineering and regenerative medicine applications involving stem cells. Our immunocytochemistry and qPCR analysis demonstrated that hESCs remained pluripotent when cultured with supplemental Mg ion dosages (up to 40 mM). We established a sensitive *in vitro* hESC culture system as a standard model for cytocompatibility studies of Mg-based and other biodegradable material, and identified a critical Mg ion dosage of 10 mM that may serve as the design guideline for safe degradation of Mg-based implants and scaffolds.

## Conclusion

We evaluated the cytocompatibility of Mg samples and the respective Mg degradation products (i.e., Mg ions and hydroxide ions) using a sensitive hESC *in vitro* model. The pre-degradation treatment for Mg did not improve H9-OCT4 hESC viability. The alkaline media condition (specifically, mTeSR®1 with a pH of 8.1) and supplemental Mg ion dosages of less than 10 mM had no adverse effect on cell morphology and did not change the coverage area of viable cell colonies as compared with the control. When hESCs were cultured with supplemental Mg ion dosages of 10 mM or greater, the hESC morphology changed from tightly packed to loosely dispersed, which led to the increase in normalized coverage area of viable hESC colonies. This critical dosage (>10 mM) of supplemental Mg ions also caused a decrease in cell counts at the endpoint of 72-hour culture. Our results indicated that hESCs continued to express pluripotency markers at supplemental Mg ion dosages of 0.4–40 mM. To advance Mg-based metallic materials for medical applications, further studies are still needed to determine the exact mechanisms of cellular responses, including not only hESCs but also other human cells.
