# Correction: An *In Vitro* Mechanism Study on the Proliferation and Pluripotency of Human Embryonic Stems Cells in Response to Magnesium Degradation

**DOI:** 10.1371/annotation/53062b94-8b47-4bdd-bde6-2725cb0a02d9

**Published:** 2014-01-06

**Authors:** Thanh Yen Nguyen, Chee Gee Liew, Huinan Liu

Figures 7 and 8 were incorrectly switched. The image currently appearing as Figure 7 belongs with the title and legend for Figure 8, and the image currently appearing as Figure 8 belongs with the title and legend for Figure 7. The titles and legends themselves are in correct order.

